# PET image segmentation using a Gaussian mixture model and Markov random fields

**DOI:** 10.1186/s40658-015-0110-7

**Published:** 2015-03-12

**Authors:** Thomas Layer, Matthias Blaickner, Barbara Knäusl, Dietmar Georg, Johannes Neuwirth, Richard P Baum, Christiane Schuchardt, Stefan Wiessalla, Gerald Matz

**Affiliations:** Institute of Telecommunications, Vienna University of Technology, Karlsplatz 13, Vienna, 1040 Wien Austria; Health & Environment Department, Austrian Institute of Technology, Donau-City-Strasse 1/2, Vienna, 1220 Wien Austria; Department of Radiation Oncology, Division of Medical Radiation Physics, Christian Doppler Laboratory for Medical Radiation Research for Radiation Oncology, Medical University of Vienna/AKH Vienna, Währinger Gürtel 18-20, Vienna, 1090 Wien Austria; Radiation Safety and Applications, Seibersdorf Labor GmbH, 2444 Seibersdorf, Seibersdorf, Austria; THERANOSTICS Center for Molecular Radiotherapy and Molecular Imaging (PET/CT) ENETS Center of Excellence, Zentralklinik Bad Berka, Robert-Koch-Allee 9, 99437 Bad Berka, Bad Berka, Germany

**Keywords:** Expectation maximization, Markov random field, Positron emission tomography, Radiotherapy, Tumor segmentation

## Abstract

**Background:**

Classification algorithms for positron emission tomography (PET) images support computational treatment planning in radiotherapy. Common clinical practice is based on manual delineation and fixed or iterative threshold methods, the latter of which requires regression curves dependent on many parameters.

**Methods:**

An improved statistical approach using a Gaussian mixture model (GMM) is proposed to obtain initial estimates of a target volume, followed by a correction step based on a Markov random field (MRF) and a Gibbs distribution to account for dependencies among neighboring voxels. In order to evaluate the proposed algorithm, phantom measurements of spherical and non-spherical objects with the smallest diameter being 8 mm were performed at signal-to-background ratios (SBRs) between 2.06 and 9.39. Additionally ^68^Ga-PET data from patients with lesions in the liver and lymph nodes were evaluated.

**Results:**

The proposed algorithm produces stable results for different reconstruction algorithms and different lesion shapes. Furthermore, it outperforms all threshold methods regarding detection rate, determines the spheres’ volumes more accurately than fixed threshold methods, and produces similar values as iterative thresholding. In a comparison with other statistical approaches, the algorithm performs equally well for larger volumes and even shows improvements for small volumes and SBRs. The comparison with experts’ manual delineations on the clinical data shows the same qualitative behavior as for the phantom measurements.

**Conclusions:**

In conclusion, a generic probabilistic approach that does not require data measured beforehand is presented whose performance, robustness, and swiftness make it a feasible choice for PET segmentation.

## Background

The determination of the tumor volume is one of the main causes for uncertainties in dosimetry [1]. When trying to assess the volume of a tumor for the sake of treatment planning in external beam radiation therapy (EBRT) or radionuclide therapy, a very common practice is to have an expert manually draw a volume of interest (VOI) on the respective positron emission tomography (PET) or single photon emission computed tomography (SPECT) image. The resulting and inevitable interobserver variations have been reported well enough for different types of cancer [2-4].

Another prevalent approach is the application of a threshold. The simplest choice for the threshold is a fixed percentage of the maximum activity concentration value [5,6]. This thresholding method has been shown to predict well for big volumes but yields large errors in case of small volumes which is attributed to partial volume effects (PVE) and moreover depends on the signal-to-background ratio (SBR). Despite its questionable scientific meaningfulness, it is still a widespread method even recommended by an international experts’ report [7]. Extensions of this method are automatic [8,9] and iterative threshold approaches [10-12]. However, the iterative thresholding method (ITM) requires a regression curve which has to be determined by phantom measurements for every specific imaging setting [13]. Considering the many dependencies of these regression curves such as the (i) manufacturer and the detector of the scanner, (ii) reconstruction algorithm, (iii) nuclide, (iv) SBR, and (v) volume of the lesion, ITM comes at a great expense in terms of work and time. Likewise, the inclusion of physical models of PET images such as a point spread function (PSF) is also dependent on some of the parameters listed above and therefore would require a considerable amount of calibration measurements.

Alternative methods such as watershed and edge detection are also sensitive to noise and different SBRs [14,15].

The aim of this work is to develop an automatic segmentation algorithm for PET images which is as generic as possible, i.e., threshold independent and therefore does not require system-specific regression curves or PSF. Additionally, the volume estimates for small objects shall be improved. Therefore, in this paper, we propose an improved statistical PET image segmentation scheme. Our scheme relies on soft class assignment instead of hard class assignments and fuzzy levels. A Gaussian mixture model (GMM) is established to obtain initial estimates of the volume of the spheres. Subsequently, a MRF is obtained by declaring Markov properties for the unobserved label vector and using a Gibbs distribution to describe neighborhood dependencies. The MRF is then used to obtain the final labeling from the initial GMM labeling vector.

## Methods

### Phantom measurements and clinical data

To evaluate the algorithm described below, a NEMA IEC Body Phantom was modified (built in-house at the Medical University of Vienna). The modified phantom differs from the original NEMA IEC Body Phantom only in the substitution of the largest sphere (37 mm) by a sphere of 8-mm diameter. Therefore, the phantom consists of a cylindrical outer body that simulates healthy tissue and six spherical inlays which represent tumor lesions with high tracer uptake. The cylindrical body was homogeneously filled with a water- ^18^F-FDG solution of low activity concentration (background (BG)) and the spherical inlays were homogeneously filled with a water- ^18^F-FDG solution of high activity concentration (foreground (FG)). A cylindrical inlay that models the lung was filled with air. The dimensions of all FG objects thus are (diameter [mm]/volume [ml]): 8/0.27, 10/0.52, 13/1.15, 17/2.57, 22/5.58, and 28/11.49. Measurements with different SBRs have been performed and are summarized in Table 1.

**Table 1:** **Measurements of the modified NEMA sphere phantom**

**FG**	**BG**	**SBR**
10.94	5.30	2.06
20.37	5.30	3.84
26.13	5.30	4.90
66.56	9.90	6.72
90.90	9.68	9.39

Activity concentration for the FG objects and for the BG object in kBq/ml and the resulting SBR.

The device in use was a Siemens Biograph 64 TruePoint PET/CT scanner (Siemens, Erlangen, Germany). In accordance with the conditions for NEMA phantom quality assurance measurements in nuclear medicine [16], the average activity concentration never exceeded 10 kBq/ml. This way, the linearity of the scanner’s noise equivalent count rate (NECR) is preserved, and the measurements of different SBRs can be compared. The acquisition was performed using emission scans of 10 min. The images were reconstructed with an iterative OSEM2D algorithm (4 iterations on 21 subsets). A preprocessing Gaussian filter of 5 mm was applied. The dimension and volume of the voxels are 4 mm ×4 mm ×3 mm and 0.048 ml, respectively. The more advanced iterative reconstruction algorithm for the Siemens scanner, TrueX (PSF), was not taken into account since recent studies recommended cautiousness with regard to its quantitative meaningfulness [17,18]. The chosen settings correspond to the clinical routine settings at the Medical University of Vienna.

In order to determine the algorithm’s performance also with regard to non-spherical objects, measurements of another in-house built phantom containing cylindrical objects have been performed. Cylinders with high activity concentration (FG) and dimensions of (diameter [mm]/volume [ml]): 10/4.08, 15/12.23, 25/37.71, and 38/103.95 have been scanned against a BG with low activity concentration and SBRs according to Table 2. All other settings were identical to the measurements of the modified NEMA IEC Body Phantom (see paragraphs above).

**Table 2:** **Measurements of the cylinder phantom**

**FG**	**BG**	**SBR**
34.20	17.40	2
42.70	10.80	4
53.20	8.90	6
75.30	9.40	8

Activity concentration for the FG objects and for the BG object in kBq/ml and the resulting SBR.

Finally, the algorithm was applied on ^68^Ga-PET data of patients suffering from disseminated neuroendocrine carcinoma that was supplied by the European Neuroendocrine Tumor Society (ENETS) Center of Excellence at the Zentralklinik Bad Berka. Lesions in the liver and lymph nodes from eight patients were segmented, and the resulting volumes were compared to the manual delineation of the experts from the ENETS Center. The image-derived SBRs of the lesions in the lymph nodes are in the order of 15, whereas for the liver they are ≤ 2.

### Statistical model

With regard to the smallest object diameter under consideration, which is only twice as large as the voxel size, PVE is a very dominating effect. It is therefore necessary to formulate partial memberships of voxels, as provided by the probability theory.

PET voxel values correspond to the activity concentration. Therefore, a PET data set comprising *N* voxels can be represented by an *N*×1 vector **x**=(*x*_1_…*x*_*N*_) whose elements are real-valued and positive, *x*_*n*_≥0, *n*=1,…,*N*. In order to describe the membership of the PET voxels in *K* distinct objects, we introduce the *K*×*N* label matrix:

(1) $$ Z =\left(\begin{array}{ccc} z_{11} \!&\! \dots \!&\! z_{1N} \\ \vdots \!&\! \ddots \!&\! \vdots \\ z_{K1} \!&\! \dots \!&\! z_{KN} \end{array} \right) = (\mathbf{z}_{1} \dots \mathbf{z}_{N}).  $$

The elements *z*_*nk*_ are binary, *z*_*nk*_∈{0,1}, with *z*_*nk*_=1 indicating that voxel *n* belongs object *k* (note that *z*_*nl*_=0 for *l*≠*k*, i.e., the row sums of **Z** equal 1).

PET image segmentation amounts to estimating the unknown label matrix **Z** given the data **x**. The proposed algorithm consists of two consecutive steps: the *coarse estimation step* fits a basic model, yielding fairly good initial estimates. These estimates are then improved in the *correction step*. The coarse estimation is performed via a modified expectation-maximization algorithm for a Gaussian mixture model (EMGMM) using information from the analysis of phantom data. The correction step compensates for overestimation of small volumes by sampling from a Gaussian MRF, using Gibbs distributions to obtain the final labeling. The Gibbs interaction parameters are chosen to act only on voxels at the boundary of two objects, i.e., voxels whose neighbors are attributed to different objects in the coarse estimation step.

For a voxel belonging to object *k*, we model *x*_*n*_ as Gaussian with mean *μ*_*k*_ and standard deviation *σ*_*k*_, $x_{n}\sim \mathcal {N}(x_{n};\mu _{k},\sigma _{k})$. Since the label specifying the object is not known, the joint likelihood function of **x** and **Z** can be written as a GMM [19,20], which is used in the coarse estimation step:

(2) $$ p(\textbf{x},\textbf{Z};\boldsymbol{\Theta}) = \prod\limits_{k=1}^{K}\prod\limits_{n=1}^{N} \left[\tau_{k}\,\mathcal{N}(x_{n};\mu_{k},\sigma_{k})\right]^{z_{nk}}  $$

Here, *τ*_*k*_ denotes the prior probabilities (normalized suchlike that $\sum _{k=1}^{K}\tau _{k}=1$) and $\boldsymbol {\Theta }=\left (\begin {array}{l} \mu _{1} \dots \mu _{K} \\ \sigma _{1} \dots \sigma _{K} \end {array}\right)$is a 2×*K* matrix containing all the Gaussian means and standard deviations.

Note that (2) does not model any statistical dependence (interaction) of different voxels. Such an interaction will be modeled only in the correction step with a Gibbs distribution [21-29] that imposes specific Markov properties on the label matrix **Z**, thus resulting in a Gaussian Markov random field (GMRF). More specifically, we use the model:

(3) $$ p(\textbf{x},\textbf{Z};\boldsymbol{\Theta},\boldsymbol{\Gamma}) = \prod\limits_{n=1}^{N} p(x_{n}|\textbf{z}_{n}) \, p(\textbf{Z};\boldsymbol{\Gamma})\,  $$

where $p(x_{n}|\textbf {z}_{n}) = \mathcal {N}(x_{n};\boldsymbol {\Theta }\textbf {z}_{n})$ is Gaussian and models the voxel activity via the mean and standard deviation (*μ*_*k*_,*σ*_*k*_)=***Θ*z**_*n*_ associated to the *k*th object. Furthermore, *p*(**Z**;***Γ***) is a Gibbs distribution [27,28] describing the interaction of the labels *z*_*nk*_:

(4) $$ p(\textbf{Z};\boldsymbol{\Gamma}) = \frac{1}{\mathcal{Z}}\prod\limits_{n=1}^{N} \exp\left(-\sum\limits_{m\in\mathcal{M}_{n}}\textbf{z}_{n}^{T}\boldsymbol{\Gamma} \textbf{z}_{m} \right).  $$

Here, $\mathcal {M}_{n}$ denotes the (first-order) neighborhood of the *n*th voxel, i.e., all voxels that share a surface with the *n*th voxel, hence $|\mathcal {M}_{n}|=6$. Furthermore, the coupling matrix is given by $\boldsymbol {\Gamma } =\left (\begin {array}{ll} 0 \!&\! \gamma \\ \gamma \!&\! 0 \end {array}\right)$.

This implies that statistical interaction is modeled only for the voxels with different labels, i.e., belonging to different objects. Finally,  is the partition function [27,28]:

(5) $$ \mathcal{Z} = \sum\limits_{\textbf{Z}} \prod\limits_{n=1}^{N} \exp\left(-\sum\limits_{m\in\mathcal{M}_{n}}\textbf{z}_{n}^{T}\boldsymbol{\Gamma} \textbf{z}_{m} \right).  $$

The specific choice of the coupling matrix ***Γ*** involves only voxels on the boundary between two objects. Since the voxels at the boundary of a FG object predominantly are part of the BG, the probability of labeling a boundary voxel as FG is decreased.

### Coarse estimation step

The FG objects of the phantom are analyzed by defining VOIs. Within the VOI, only the FG (*k*=1) and the BG (*k*=2) need to be distinguished (hence, *K*=2, *cf*. (1)). The problem of simultaneously estimating the labeling matrix **Z** and the parameter matrix ***Θ*** can be formulated in terms of the expectation-maximization (EM) algorithm (see [19-21,30-37] for further details and application):

(6) $$ \hat{\boldsymbol{\Theta}}^{(i+1)} = {\underset{\boldsymbol{\Theta}}{\text{arg max}}}\; E_{\mathbf{Z}|\mathbf{X};\hat{\boldsymbol{\Theta}}^{(i)}} \left\{ \log p(\mathbf{x},\mathbf{Z};\boldsymbol{\Theta})\right\},  $$

where *p*(**x,Z**;***Θ***) is defined in (2).

**Figure 1:**
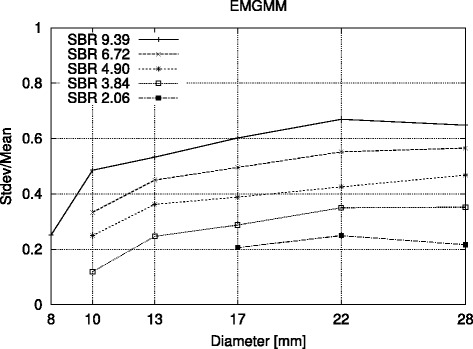
**Normalized standard deviation versus object diameter.** Plot of normalized standard deviation $\boldsymbol {\tilde \sigma _{2}}$ for BG (estimated using the conventional EM algorithm) versus object diameter at several SBRs with the remodeled NEMA phantom (including morphologically not connected objects).

Unfortunately, small FG objects consist only of a few voxels and hence represent poor statistical ensembles for the EM algorithm, resulting in inaccurate parameter estimates. Furthermore, in an FG object, the number of PVE voxels tends to be equal to or even larger than the number of pure FG voxels, causing the PVE voxels to be classified as FG (note that the statistical ensemble for the BG is rather large and therefore reliable). Consequently, we observe an SBR-dependent overestimation of the FG volumes which in many cases are not even morphologically connected objects. For the BG, the normalized standard deviation $\tilde \sigma _{2} = \frac {\sigma _{2}}{\mu _{2}}$ amounts to 10*%* uniformly over all object sizes and SBRs. In contrast for the FG, $\tilde \sigma _{1}$ depends significantly on the object size and SBR as can be seen in Figure 1.

In order to avoid the problem with the increased FG standard deviation, we use a modified EM algorithm in which *σ*_1_ is set equal to *σ*_2_. The resulting EM starts with an initial, reasonable guess for the means and standard deviations of FG as well as BG and iterates Bayesian estimation and maximization as follows:

- The conditional expectation $\bar {z}_{\textit {nk}}^{(i)} = E_{z_{\textit {nk}}|x_{n};\theta _{k}^{(i)}}\{{z}_{\textit {nk}}\}$ is updated as:
  (7) $$ \bar{z}_{nk}^{(i)} = \frac{\tau_{k}^{(i)}\,\mathcal{N} \left(x_{n};\mu_{k}^{(i)},\sigma_{k}^{(i)}\right) }{\sum_{k=1}^{2} \tau_{k}^{(i)}\, \mathcal{N}\left(x_{n};\mu_{k}^{(i)},\sigma_{k}^{(i)}\right) }.  $$
- The parameter updates read:
  (8) $$ \begin{aligned} \tau_{k}^{(i+1)} & = \sum\limits_{n=1}^{N} \frac{\bar{z}_{nk}^{(i)}}{N}, \\ \mu_{k}^{(i+1)} & = \frac{\sum\limits_{n=1}^{N} x_{n} \bar{z}_{nk}^{(i)}}{\sum\limits_{n=1}^{N} \bar{z}_{nk}^{(i)} },\\ \sigma_{2}^{(i+1)} & = \sqrt{\frac{\sum\limits_{n=1}^{N} (x_{n} - \mu_{2}^{(i+1)})^{2} \bar{z}_{n2}^{(i)} }{\sum\limits_{n=1}^{N} \bar{z}_{n2}^{(i)}}},\\ \sigma_{1}^{(i+1)} & = \sigma_{2}^{(i+1)}. \end{aligned}  $$
- A soft labeling is achieved by using the conditional probabilities $\bar {z}^{(i)}_{\textit {nk}}$:
  (9) $$ \hat{z}^{(i)}_{nk} = \bar{z}^{(i)}_{nk}.  $$

Due to the usage of a prior probability for **Z**, the resulting parameter estimations are done according to weighted averages.

### Correction step

To improve the coarse estimates obtained via the modified EM algorithm operating on the Gaussian mixture model, a second labeling problem for **Z** is formulated on the GMRF (3) via a maximum *a posteriori* (xMAP) estimate:

(10) $$ \hat{\mathbf{Z}}= {\underset{\mathbf{Z}}{\text{arg max}}}\; \left\{ p(\mathbf{x,Z};\boldsymbol{\Theta},\gamma) \right\}.  $$

We solve (10) numerically by Metropolis sampling according to the procedure described below using the labeling obtained in the first coarse estimation step as initialization with its parameters held fixed to obtain a refined segmentation.

New labeling proposals are generated by changing local values of **Z** (i.e., changing a FG voxel to BG or vice versa). Assume $\mathbf {z}_{n}^{(i+1)}$ is the voxel label that has been changed and let $(\mu _{k},\sigma _{k}) = \boldsymbol {\Theta }\mathbf {z}_{n}^{(i+1)}$; furthermore,

(11) $$ P^{(i+1)}_{n} \propto \exp\left(- \sum\limits_{m\in\mathcal{M}_{n}} \mathbf{z}_{n}\boldsymbol{\Gamma}\mathbf{z}_{m} - \frac{(x_{n}-\mu_{k})^{2}}{2{\sigma_{k}^{2}}}\right)  $$

denotes the local conditional probability of the labeling proposal. If $P^{i+1}_{n} \ge {P^{i}_{n}}$, the new label is accepted. If $P^{i+1}_{n} < {P^{i}_{n}}$, the proposal labeling is accepted with probability $P_{n}^{i+1}/{P_{n}^{i}}$ and rejected with probability $1-P_{n}^{i+1}/{P_{n}^{i}}$.

After a predefined number *L* of full samples (labeling configurations where each voxel is processed once) have been obtained in this manner, the final labeling is computed by forming the arithmetic mean of all accepted full proposal labeling, i.e.,

(12) $$ \hat{\mathbf{Z}}=\frac{1}{L}\sum\limits_{l=1} \mathbf{Z}^{(l)}.  $$

### Threshold methods

For clinical applications of volume determination in PET, values of 36% to 42% of the maximum value have been proposed as local threshold [5]. In this context ‘local’ means that the threshold is calculated using the VOI and not the whole image.

With ITM, regression curves are needed, i.e. measurements of a phantom at different SBRs. The percentage threshold yielding the true volume is calculated as function of the volume and the SBR. Using ITM for automatic segmentation, an initial threshold is applied to a VOI followed by the determination of the resulting volume (*V*) and SBR. With those values, the corresponding threshold from the regress function *%*Thr=*f*(*V*,SBR) is further applied to the VOI, resulting in an iterative update scheme. The algorithm stops when the deviation between two iterations is ≤0.1*%*.

For purposes of evaluation and comparison, we applied the EMGMM, the proposed GMRF algorithm, and the aforementioned thresholding methods to the phantom measurements as well as the clinical data discussed in the ‘Phantom measurements and clinical data’ section. A graphical user interface was built by the object-oriented programming language IDL to visualize and process the data and to draw VOIs around each FG object. To investigate whether the results depend on the VOI size, we used VOIs consisting of 14×14×20, 14×14×40, 22×14×20, and 22×14×40 voxels.

## Results

**Figure 2:**
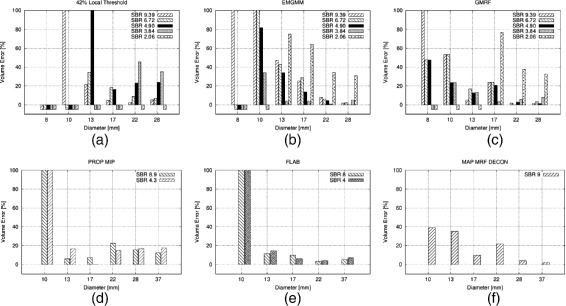
**Volume error achieved by various methods for different NEMA sphere diameters, and SBRs.** **(a)** 42% thresholding, **(b)** EMGMM, **(c)** GMRF, **(d)** statistical approach acting on MIP [40], **(e)** fuzzy locally adaptive Bayesian approach [38], and **(f)** maximum *a posteriori* GMRF approach with subsequent deconvolution [39]. **(a)**, **(b)**, and **(c)** use VOIs of 14×14×20 voxels; **(d)**, **(e)**, and **(f)** were copied visually from [38,40] and [39]. Note that the diameter ranges vary in **(a-f)**.

**Figure 3:**
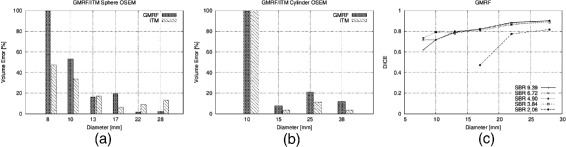
**Volume error achieved by GMRF versus ITM averaged over different SBRs.** **(a)** NEMA sphere OSEM reconstructions. **(b)** Cylinder phantom OSEM reconstructions. **(c)** DSC between GMRF and ground truth from CT scan for spherical objects.

**Figure 4:**
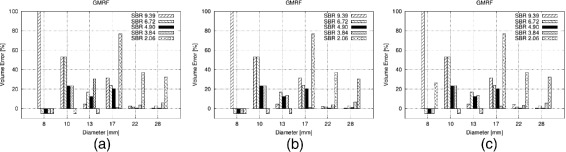
**Volume error for different NEMA sphere diameters and SBRs obtained with GMRF on VOIs of size (a)** ***14×14×40*** **, (b)** ***22×14×20*** **, and (c)** ***22×14×40*** **voxels.**

Figures 2a,b,c,d,e,f, 3a,b, and 4a,b,c in this section show the relative volume error (in percent) for the FG objects versus their diameter. For each diameter, several bars are drawn to account for the different SBRs. FG objects are considered as detected if the segmentation yields two morphological connected objects, i.e., a FG object surrounded by BG volume. If a FG objects is not detected according to this definition, this is indicated graphically by a volume error of −5*%*. Furthermore, the dice similarity coefficient (DSC) is presented for the comparison of GMRF vs. ground truth of the CT as well as for the clinical Query: Please shorten the title for Figure 4 up to 15 words only to comply with the journal instruction. data.

Furthermore, Tables 3 and 4 give an overview of the number of detected FG objects with regard to each segmentation strategy and SBR.

**Table 3:** **Number of FG objects detected by 36% and 42% thresholding, ITM, EMGMM and GMRF**

**SBR**	**36%**	**42%**	**ITM**	**EMGMM**	**GMRF**
2.06	0	0	0	4	3
3.84	0	2	4	5	5
4.90	3	4	4	5	6
6.72	4	4	5	5	6
9.39	5	5	6	6	6

The maximum amount of detectable FG objects is 6.

**Table 4:** **Number of FG objects (cylinder phantom) detected at different SBRs by the ITM and the GMRF**

**SBR**	**ITM**	**GMRF**
2	0	0
4	2	3
6	3	3
8	4	4

### Threshold methods

Figure 2a shows the results for local thresholding with a threshold of 42%. Clearly, for small FG objects, the activity concentration is underestimated due to PVE which leads to large volume errors. It is seen that for all FG objects the error increases with decreasing SBR. This also applies to ITM whose results are depicted in Figure 3a,b. Here, the volume error is averaged over the SBR in order to keep the amount of results readily comprehensible. No threshold method can detect FG objects at an SBR of 2.06. Furthermore only the ITM is able to detect the smallest FG object of 8-mm diameter and only at the biggest SBR of 9.39.

### EMGMM and GMRF

**Figure 5:**
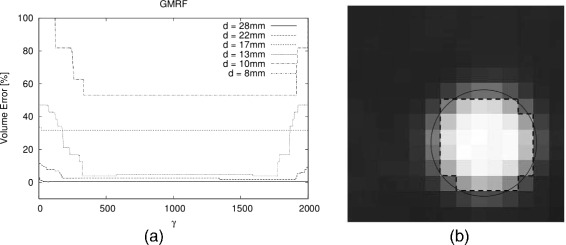
**Volume error and segmentation result.** **(a)** Volume error achieved by GMRF for different NEMA sphere diameters at SBR=9.39 drawn over the parameter ***γ***. **(b)** Segmentation result of the 28-mm sphere at SBR=9.39 after application of the GMRF (dashed lines) and ground truth from CT (black circle).

The correction step is performed with *γ*=1,000 since the GMRF algorithm produces stable results in this region (see Figure 5a). Since the coarse estimation step provides initial values that are already close to the energetic minimum, the label configurations attain equilibrium after the first ten samples which get discarded. For *L*> 70, no change in results is observed. Figure 5b shows the GMRF segmentation result of the 28-mm sphere at SBR=9.39 as compared to the ground truth derived from the CT. For this, the center of the sphere was identified in the high-resolution CT and the known radius of the real phantom was plotted.

The results of the proposed GMRF method and its EMGMM initialization are shown in Figure 2c,b, respectively, for VOIs comprising 14×14×20 voxels. With regard to both spheres and cylinders, EMGMM as well as GMRF achieve a much better detection rate of FG objects than the threshold methods (see Tables 3 and 4). In particular, GMRF detects all six spherical FG objects at SBRs above 4.90 as well as the three larger spheres at SBR 2.03. The total detection rate score of the GMRF with 26/30 and 10/16 for spheres and cylinders, respectively, clearly supersedes the ITM with 19/30 and 9/16.

Concerning volume segmentation, GMRF achieves lower errors than the fixed threshold approaches for almost all combinations of SBR and diameter. In a direct comparison of GMRF and ITM (see Figure 3a,b), ITM performs better on smaller spheres while GRMF is better on larger ones. For cylindrical objects, ITM shows slightly smaller errors for diameters ≥15 mm. Figure 3c shows the DSC which was calculated for the phantom with spherical objects as a comparison of GMRF segmentation vs. ground truth from the CT. For objects with diameters ≥13 mm and SBR ≥3.84, the DSC ≥0.8.

### Comparison to threshold-independent algorithms

Figure 2e,f shows the results obtained by Hatt and co-workers using a fuzzy locally adaptive Bayesian (FLAB) algorithm [38] and by the maximum *a posteriori* estimation for a GMRF with deconvolution (MAP-MRF-DECON) designed by Gribben et al. [39], respectively (the data has been manually copied from the above cited publications). For further comparison, a statistical approach by Dewalle-Vignion and co-workers acting on maximum intensity projections (PROP MIP) [40] including the fuzzy c-means technique is shown in Figure 2d. While those papers also performed NEMA phantom measurements, FG objects with different diameter ranges were used. In particular, none of the previous works used a FG object as small as 8 mm of diameter or an SBR as low as 2.06.

Comparing GMRF with the MAP-MRF-DECON algorithm, the former performs better for some diameters and worse on others. However, a systematic comparison is not possible since [39] provides results only for the rather large SBR of 9. PROP MIP and FLAB yield quite accurate volumes for spheres greater than 10 mm but performs poorly when it comes to spheres smaller than 13-mm diameter (the smallest sphere used in [40] and [38] is 10 mm). At such diameters, GMRF approaches have to be preferred due to their better performance.

### VOI dependencies of GMRF

Finally, Figure 4 shows the results obtained with GMRF for different VOI sizes. As can be seen for all spheres with diameter ≥10 mm, the volume errors are constant over the chosen VOI range with regard to all SBRs. Concerning the 8-mm sphere, no correlation can be found between the detection rate and VOI size. However, if detected, the volume error is also constant over the VOI range.

### Clinical data

With regard to the clinical data, GMRF’s segmentation of the metastases in the lymph nodes yields volumes which are 15% to 20% bigger than the manual delineation. This value is constant over a volume range down to 3 ml wherefrom the overestimation gets bigger. Taking into account that the SBR for the lesions in the lymph nodes is 15, this behavior is very similar to the measurements of the NEMA spheres at higher SBRs. Likewise, the GMRF’s segmentation of lesions in the liver with very small SBRs (≤2) shows a volume which is constantly smaller by 30*%* and therefore reflects very well the behavior of phantom measurements at very low SBRs.

**Figure 6:**
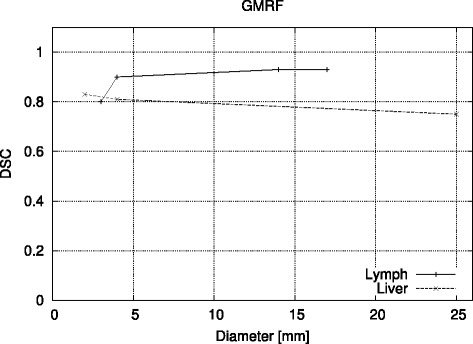
**DSC between manual delineation and GMRF for** ^**68**^ **Ga-PET of neuroendocrine metastases in the liver and lymph node.**

Figure 6 shows the DSC between the manual experts’ delineation and the resulting volumes when GMRF is applied to the clinical data. For metastases in the lymph nodes, the mean DSC is 0.89 and for the liver 0.80.

## Discussion

Forcing equal standard deviations for BG and FG as discussed in the ‘Coarse estimation step’ section helped to reduce the segmentation error. While this approach appears somehow arbitrary, it can be qualitatively understood as follows: Figure 1 shows the SBR diversification for the normalized standard deviation $\tilde \sigma _{1}$ of FG. For higher SBR, $\tilde \sigma _{1}$ and therewith the volume estimates increase because the PVE voxels get encompassed by the FG object. By setting *σ*_1_=*σ*_2_, the PVE voxels are interpreted as BG such that in the subsequent estimate *σ*_2_ increases. As a result, also *σ*_1_ increases and parts of the PVE voxels are re-assigned to FG, in turn lowering *σ*_1_. This interplay causes a conversion towards the solutions shown in the ‘Results’ section.

Likewise, the value *γ*=1,000 was introduced without further explanation. Numerical calculations have shown that values for *γ* between 500 and 1,500 yield stable non-zero volume estimates for the FG objects instead of downgrading the volumes until they vanish (increasing the amount of neighborhood voxels $|\mathcal {M}_{n}|$ leads to zero volume solutions). Since the coarse estimation step already yields good initial label estimates with a large value of *γ* as defined above, the system reaches equilibrium after the first three full samples have been obtained. Storing more than 70 subsequent configurations at equilibrium according to (12) does not change the solutions presented here.

When comparing the proposed GMRF method and its initializing EMGMM (see Figure 2c,b), it becomes apparent that the proposed correction step enforced in the GMRF decreases the volume error for small FG objects as intended by the choices described in the ‘Correction step’ section. This emphasizes the usefulness of MRFs as a powerful tool regarding PET image segmentation, especially in the case of small objects. In this sense, the findings presented here confirm the work of Gribben et al. [39] that also uses MRFs. Nonetheless, it can be seen in Figure 2 that the initialization step is not fully corrected for SBR diversification, especially for small FG objects. Acting with Gibbs distributions on this solution as described above is not sensible to different SBRs. Therefore, future work should aim for compensating SBR dependencies in the correction step.

The results of the GMRF also show that for spheres with diameter ≥13 mm, the cold-wall effect can be accounted for. For smaller spheres, the cold-wall effect gets noticeable as for all segmentation approaches in the literature [8-40].

The choice of the comparison methods shall be briefly outlined. Despite the existence of iterative threshold approaches (see the ‘Background’ section), fixed thresholds are very common in clinical practice. As we can see in the ‘Results’ section, GMRF clearly outreaches the fixed threshold approaches both in the detection rate and volume error, a result not very surprising. However, the fact that GMRF also outperforms ITM on detection rate and produces similar results with regard to the volume error and DSC is remarkable all the more, as GMRF uses no *a priori* knowledge whatsoever whereas ITM has a the luxury of a validated calibration curve. Taking into account the dependencies of these regression curves (see the ‘Background’ section) and as a consequence thereof the need of not only one but many phantom calibration measurements, GMRF seems a much more practical approach for clinics since it is equally reliable but can be directly applied for different systems, nuclide, reconstructions, etc. Furthermore, an ITM only can realize a segmentation of whole voxels whereas the inclusion of probability theory as in our approach can assign partial classifications to voxels which is inevitable in the case of small volumes. The comparison with the threshold-independent algorithms also shows that the algorithm in this work performs equally well for larger volumes and even shows improvements for small volumes and SBRs.

When discussing the segmentation of the clinical data, one has always to recall that unlike the real volume of the phantoms the manual delineation does not represent the ground truth, i.e., it can only be a comparative analysis. Systematic overestimation as well as underestimation of the tumor volume has an extensive impact on radiation treatment planning. In practice, the so-called planning target volume (PTV) is an extension of the visible tumor taking into account microscopic spread as well as uncertainties in dose delivery and patient positioning. Therefore, a systematic overestimation will partially coincide with the PTV and be of no severe consequences as long as the overlap is not too big and thus causes widespread damage to the surrounding healthy tissue. In contrast, a systematic underestimation involves the danger of not irradiating the entire tumor and thus enhances the chances for relapse.

A mean DSC of 0.9 for high SBRs, the robustness of the results over a large volume range, and the resemblance to the results of the phantom measurements concerning the SBR dependency make the GMRF a suitable candidate for future studies that encompass patient data of higher quantity and larger diversity. Moreover, to study realistic images with inhomogeneous activity distributions and be in possession of the ground truth, simulated images have to be included as also done by various authors [11,40-43].

The proposed GMRF produces stable results for different lesion shapes (see Figure 3) and therefore underlines its potential for future clinical use. Another benefit of the proposed algorithm is its fast convergence. With the termination condition:

(13) $$ |\mu_{k}^{(i+1)} - \mu_{k}^{(i)}| < 0.01, \quad k=1,2,  $$

the number of EMGMM iterations in the case of detection did not exceed 50. Given a standard laptop (dual core 2×1.8 GHz), the overall processing time stays well below 1 s even for the case of large VOI. In this time frame, it is even feasible to use the algorithm several times to repeat the processing with subVOIs to improve the detection rate for small objects.

## Conclusions

Segmentation of PET data remains a very challenging issue since pertinent algorithms are very sensitive to a variety of parameters. The aim of this work was to investigate the aspects that were not sufficiently covered in the literature so far, namely the impact of the SBR, the segmentation of objects with a diameter smaller than 10 mm, and the waiver of any *a priori* knowledge such as a regression curve. Therefore, phantom measurements with spherical as well as non-spherical objects with SBRs ranging from 2 to 9.36 have been evaluated, including a FG sphere with a diameter of 8 mm. Additionally, lesions from clinical data have been segmented.

Combining an EMGMM with MRFs, taking advantage of Gibbs distributions to describe neighbor dependencies, results in a significant decrease of the overestimation of small volumes on the one hand and on the other hand yields vanishing volume errors in the case of bigger objects and high SBRs. The proposed algorithm has advantages over threshold methods and can be applied to any PET data, not requiring any system-specific regression curves in order to account for the given nuclide, manufacturer, or reconstruction algorithm. The comparison with experts’ manual delineations on clinical images shows the same qualitative behavior as for the phantom measurements. In connection with its rapidness and the insensitivity towards reconstruction algorithm and lesion shape, the proposed algorithm is a suitable choice for PET segmentation, even though there is still room for improvement in future work.
